# Cu,Zn Superoxide Dismutase Genes in *Tribolium castaneum*: Evolution, Molecular Characterisation, and Gene Expression during Immune Priming

**DOI:** 10.3389/fimmu.2017.01811

**Published:** 2017-12-18

**Authors:** Kevin Ferro, Diana Ferro, Francesca Corrà, Rigers Bakiu, Gianfranco Santovito, Joachim Kurtz

**Affiliations:** ^1^Institute for Evolution and Biodiversity, University of Münster, Münster, Germany; ^2^Department of Biology, University of Padova, Padova, Italy; ^3^Department of Aquaculture and Fisheries, Faculty of Agriculture and Environment, Agricultural University of Tirana, Tirana, Albania

**Keywords:** superoxide dismutase, *Bacillus thuringiensis*, immune priming, *Tribolium castaneum*, innate immunity, reactive oxygen species

## Abstract

The production of reactive oxygen species (ROS) is a normal consequence of the aerobic cell metabolism. Despite their high and potentially detrimental reactivity with various biomolecules, the endogenous production of ROS is a vital part of physiological, immunological, and molecular processes that contribute to fitness. The role of ROS in host–parasite interactions is frequently defined by their contribution to innate immunity as effectors, promoting parasite death during infections. In vertebrates, ROS and antioxidant system enzymes, such as superoxide dismutase (SOD) are also involved in acquired immune memory, where they are responsible for T-cell signalling, activation, proliferation, and viability. Based on recent findings, ROS are now also assumed to play a role in immune priming, i.e., a form of memory in invertebrates. In this study, the potential involvement of Cu,Zn SODs in immunity of the red flour beetle *Tribolium castaneum* is described for the first time, applying an approach that combines an *in silico* gene characterisation with an *in vivo* immune priming experiment using the Gram-positive entomopathogen *Bacillus thuringiensis*. We identified an unusually high number of three different transcripts for extracellular SOD and found that priming leads to a fine-tuned modulation of SOD expression, highlighting the potential of physiological co-adaptations for immune phenotypes.

## Introduction

Reactive oxygen species (ROS) are oxygen derivatives commonly produced in eukaryotic cells as a consequence of the aerobic metabolism. One of the main sites of formation are mitochondria, where ROS are produced by interaction between molecular oxygen (O_2_) and the electron transport chain in the inner membrane (1). A physiological concentration of ROS is not harmful, but rather necessary for cell viability (2). For example, ROS have a key role in intracellular signalling, resulting in modulation of cellular processes such as stem cell proliferation and differentiation, autophagy, and cellular ageing (3, 4). In humans, the perturbation of ROS homoeostasis can lead to various pathologies, including neoplastic proliferation, Parkinson, Alzheimer’s and cardiovascular diseases (5). ROS are not only produced by endogenous processes, but also by numerous exogenous factors such as exposure to pathogens and xenobiotics, UV radiation as well as variation in temperature (6–10). In order to keep ROS production and elimination in balance, organisms evolved a complex molecular machinery, the antioxidant system. The components of the antioxidant system can be classified in enzymatic molecules [e.g., superoxide dismutase (SOD), catalase, glutathione (GSH) peroxidases, peroxiredoxins, and methionine sulphoxide reductase] and non-enzymatic ones (e.g., GSH, vitamin C, vitamin E, phytochelatins, and metallothioneins) (1, 7, 10–13). These molecular components of the cell have been conserved throughout deep evolutionary time in all eukaryotic organisms, from yeast to vertebrates (14–17).

The production of ROS is a prominent feature of innate immune responses. For example, after oral infection, insect hosts can heavily depend on an increase of ROS in the gut lumen, often in concert with a local up-regulation of antimicrobial peptides (AMPs) (6, 18–20). Moreover, during bacterial antigen recognition by phagocytes, both ROS and nitrogen oxide are produced as a consequence of phagocytosis, in order to act as cytotoxins and second messengers (14). In vertebrate acquired immunity, ROS take an active role in the determination of T-cell states (21) and moreover, T cells are also affected by changes in the regulation of the antioxidant system within the phagocytic cells, especially non-enzymatic ROS scavengers such as GSH (11, 22). Additionally, enzymatic antioxidant system components, such as SOD enzymes, are up-regulated during T-cell death and activation, as a response to increased mitochondrial superoxide (•O_2_^−^) formation (23). In summary, the role of ROS for infection dynamics in general, and host immune modulation in particular, is essential.

Although innate immunity is a feature of virtually all known multicellular organisms, acquired immunity (i.e., plastic immune responses based on specific memory) is mainly considered a hallmark of vertebrate species. However, recent studies demonstrated that invertebrates are also capable of mounting alternative forms of adaptive immune responses despite lacking features of classical vertebrate immunity (24, 25). Amongst others, one prominent example is the red flour beetle *Tribolium castaneum*. In this model organism, enhanced protection after repeated exposure to the same bacterial pathogen was demonstrated for septic as well as oral infection routes (26, 27), and could even be transferred to offspring (28, 29). This phenomenon is referred to as immune priming, and it has now been reported in a wide range of invertebrate species [for review see Ref. (24)], including copepods, water fleas, cockroaches, snails, shrimps, and mealworm beetles (25, 30). Although immune priming in these species is well studied, the molecular machinery underpinning immune priming is not well understood. Only recently the identification and characterisation of immune priming-related pathways has begun (24, 31–33). For example, the involvement of AMDs, as well as phagocytosis, in immune priming has been experimentally described in *Drosophila melanogaster, Tenebrio molitor*, and *Galleria mellonella* (34–37).

In *T. castaneum*, the analysis of transcriptomic data from two populations of *T. castaneum*, infected with the entomopathogen *Bacillus thuringiensis* by septic wounding, emphasised the importance of an up-regulation of transcription in genes with a predicted oxidative function for the systemic immune response (18). Another recent transcriptomic study in the same system, but using oral priming by exposure to bacteria-free spore culture supernatants showed that ROS genes were already up-regulated after priming, even when not followed by infection with live spores (38). This finding encouraged us to explore the ROS metabolism of *T. castaneum* during septic priming with this micro-parasite, and furthermore the potential role of ROS-detoxifying genes in invertebrate immune memory. Since comprehensive information about antioxidant system genes in *T. castaneum* is not available, we decided to begin our investigation with the first and most important line of defence against ROS, the SOD genes (7, 12).

Superoxide dismutase (SOD EC 1.15.1.1) is a ubiquitous family of metallo-enzymes that catalyse the dismutation of •O_2_^−^ to H_2_O_2_ and are considered ancient enzymes, due to their unique evolutionary history (39, 40). SODs can be classified on the basis of the metal present in the catalytic core that is used for the dismutation process. In eukaryotic cells, three SOD types have been identified: manganese SOD (Mn SOD or SOD2), localised in mitochondria, and two Cu,Zn SODs, named intracellular SOD (IC-SOD or SOD1) and extracellular SOD (EC-SOD or SOD3), with respect to their cellular localisation (41, 42). In the present study, we focussed on the characterisation of Cu,Zn-SOD genes combining bioinformatics, biomolecular, and biochemical techniques, in order to gain first data on the evolutionary history of Cu,Zn SODs in *T. castaneum*. Moreover, for the first time, we characterised the involvement of Cu,Zn SODs in physiological responses to immune priming, studying gene expression at both transcriptional and translational levels, in a full-factorial priming and challenge experiment using *B. thuringiensis*.

## Materials and Methods

### Animals and Infection Protocol

Larvae of *T. castaneum* were obtained from our laboratory strain CRO1 (43). For oviposition, around 200 adults were transferred into glass jars containing fresh flour with 5% yeast. The animals were then kept for 1 day in a photoperiodic rhythm of 12:12 (D:L), at a temperature of 30°C and 70% humidity. After 11 days, a total of 960 larvae were collected and individualised into 96-well plates. In our experiment, we carried out controlled immune priming (first exposure) and challenge (second exposure) treatments using the bacteria *B. thuringiensis* strain DZSM 2046 [denoted “Bt1” in Roth et al. (26)]. Bacterial cultures were grown as previously described (26).

For the immune priming, the bacteria suspension was first adjusted to a concentration of 1 × 10^9^ cells × mL^−1^ and subsequently heat-killed in a Thermomixer comfort for 30 min at 95°C [heat-killed Bt1 (hkBt1)]. For the challenge, we used live bacteria at the concentration of 5 × 10^5^ cells × mL^−1^.

The appropriate bacteria concentrations used in this study were determined on the basis of preliminary experiments conducted in our laboratory, aiming at a priming concentration that leads to enhanced survival upon following challenge, and the lowest concentration that still induces mortality upon challenge, respectively (data not shown).

The injections were performed using the Nanoject II™ Auto-Nanoliter Injector equipped with two-step pulled, cut, and backfilled glass capillaries. Every injection was performed under a binocular microscope and every larva was injected with 18.4 nL of either a bacteria suspension or PBS. For priming, 15-day old larvae were injected laterally between the second- and third-last segment. For challenge, 19-day old larvae were injected dorsally between the head and the first segment to prevent any unwanted effects of repeated wounding at the same location. After each injection, larvae were transferred into 96-well plates filled with flour and 5% yeast for subsequent monitoring. One-day past infection the survival of all larvae was scored, the survivors collected and flash frozen in liquid nitrogen. Samples were stored at −80°C until use. The time point for sampling was chosen based on previous studies, that describe the survival curve of *T. castaneum* upon challenge with Bt1, in order to ensure that the infected individuals were not moribund (26, 32). Three independent experiments were performed for each priming/challenge combination.

### Primer Design, RNA Extraction, cDNA Synthesis

The latest version of the *T. castaneum* genome (Assembly 5.2^1^) was examined for genomic regions that might code for Cu,Zn-SOD enzymes. We identified three genomic regions for which the following gene sequences where annotated as predicted: TC007011, TC011676, and TC011675 that we named *tc-soda, tc-sodb*, and *tc-sodc*, respectively. The analysis of previously obtained RNAseq data from the Cro1 population (18) revealed that *tc-sodb* could potentially be transcribed in two variants: Tc-SODb1 and Tc-SODb2. We used the predicted sequences based on RNAseq data to design specific primers and confirm their expression, in order to study their gene transcription after immune priming and challenge. The primers were designed in the conserved domains, across exon–exon junctions, and their sequences were analysed with IDT Oligo analyser.^2^ As we used a low challenge dose of Bt1 to study SOD expression modulation, we verified the infection status by measuring the expression level of the AMP *attacin 2* (*atta2*), which is known to respond to septic infection with *B. thuringiensis* (18). Primers chosen for real-time qPCR are shown in Table S1 in Supplementary Material. Total RNA was isolated from a pool of nine larvae for each priming/challenge combination and each experimental replicate with peqGOLDTriFast™ reagent (PEQLAB) according to the manufacturer’s protocol. Total RNA obtained was purified with NaAc 5 M to remove glucidic contaminants and the quantification was performed using the ND-1000 spectrophotometer (Thermo Scientific). The first strand of cDNA was reverse-transcribed at 42°C for 1 h from 1 µg of total RNA in a 20 µL reaction mixture containing 1 µL of RevertAid Reverse Transcriptase (ThermoFisher Scientific) and 0.5 µL oligodT Anchor primer.

### Quantitative Real-time PCR (qRT-PCR)

Quantitative real-time PCR analyses were performed in a final volume of 10 µL, with 1–10 ng of cDNA, 1 × KAPA™ SYBR Green PCR Master mix, 0.5 µM for each primer. A dissociation curve was used to confirm the specificity of the amplicon. We verified the efficiency of the primers by drawing standard curves for target genes (amplification efficiency >1.9). qRT-PCR reactions were performed in duplicates in a PCR system LightCycler^®^ 480 (Roche) as described in previous experiments (44). Thermal cycling conditions were as follows: 2 min denaturation at 95°C followed by 38 cycles for 25 s denaturation at 95°C, 1 min annealing and elongation at 60°C, and a final 3 min elongation at 72°C. The 2-ddCt (relative quantification) was used to calculate the relative expression ratio (45) with respect to the house-keeping gene (rp49, ribosomal protein). This method defines the change in expression of a nucleic acid sequence (target) in test samples relative to the same sequence in a calibrator sample (naive/naive), when the target gene is expressed at its lowest levels. Random samples of amplicons resulting from the RT-qPCR reaction were purified with Spin PCRapace (Stratec biomedical MSB^®^) and sequenced with the 3130xl Genetic Analyzer (Applied Biosystems) to confirm the specificity of the designed primers.

### Sequence Analysis, *In Silico* Promoter Studies, and Molecular Phylogeny

The complete protein sequences of SODs from *T. castaneum* were aligned to other confirmed metazoan sequences, using the Muscle programme in order to study their level of conservation (46). Considering that some metazoan IC-SODs and EC-SODs have already been structurally characterised *via* X-ray crystallography, we employed a comparative modelling approach to study *T. castaneum* SODa, SODb, and SODc folding (for which no experimental structure is currently available). In this work, homology protein modelling was carried out with SwissModel bioinformatic tools^3^ (47). A 2 kbp region upstream of the respective coding sequences of the *sod* genes was analysed with a handmade pipeline designed in Python 3.0 to identify putative transcription factor binding sites such as antioxidant responsive element (ARE), metal responsive element, and xenobiotic responsive element (XRE) sites, according to previous studies (7, 11, 12). The sequences used for the molecular phylogeny in this paper are summarised in Table S2 in Supplementary Material along with their GenBank accession numbers. cDNA and amino acid sequences were aligned with T-Coffee (48). Analyses were performed using 88 candidate models and three types of criteria: Akaike information criterion (AIC), corrected AIC, and Bayesian information criterion. For carry out the statistical selection of the best-fit model of nucleotide substitution and protein evolution jModelTest 2.0 and ProtTest 3 were used, respectively (49).

### SOD Activity Assay

The larvae that survived challenge, and that were not used for molecular biology analyses, were homogenised as previously described (12). Homogenates were centrifuged at 48,000×*g* for 60 min at 4°C, and the resulting supernatants were used for SOD activity quantification. We used the method described by Fridovich (41), which is based on the inhibition of the reduction of nitro blue tetrazolium (NBT) by •O_2_^−^ produced *via* riboflavin photo reduction. One unit of SOD activity was defined as the amount of enzyme required for 50% inhibition of NBT conversion. Data refer to total protein concentrations, assayed by the Lowry method (50, 51) with some modifications.

### Statistical Analysis

Survival data were analysed using multiple pairwise Wilcoxon tests across treatments in R Version 3.3.2.^4^ qRT-PCR and enzyme activity data were expressed as average of three independent experiments and statistical analyses were performed with the PRIMER statistical programme. ANOVA for different groups was followed by the Student–Newman–Keuls test to assess significant differences between the treatments (*p* < 0.05).

## Results

### Gene Architecture of *T. castaneum* Cu,Zn-SOD Genes

In the *T. castaneum* Genome Database (scaffold ids 34, 67, and 56), we identified three sequences putatively coding for members of the Cu,Zn SOD family. We named the respective genes *tc-soda* (OGS-ID TC007011), *tc-sodb* (OGS-ID TC011676), and *tc-sodc* (OGS-ID TC011675).

**Figure 1:**
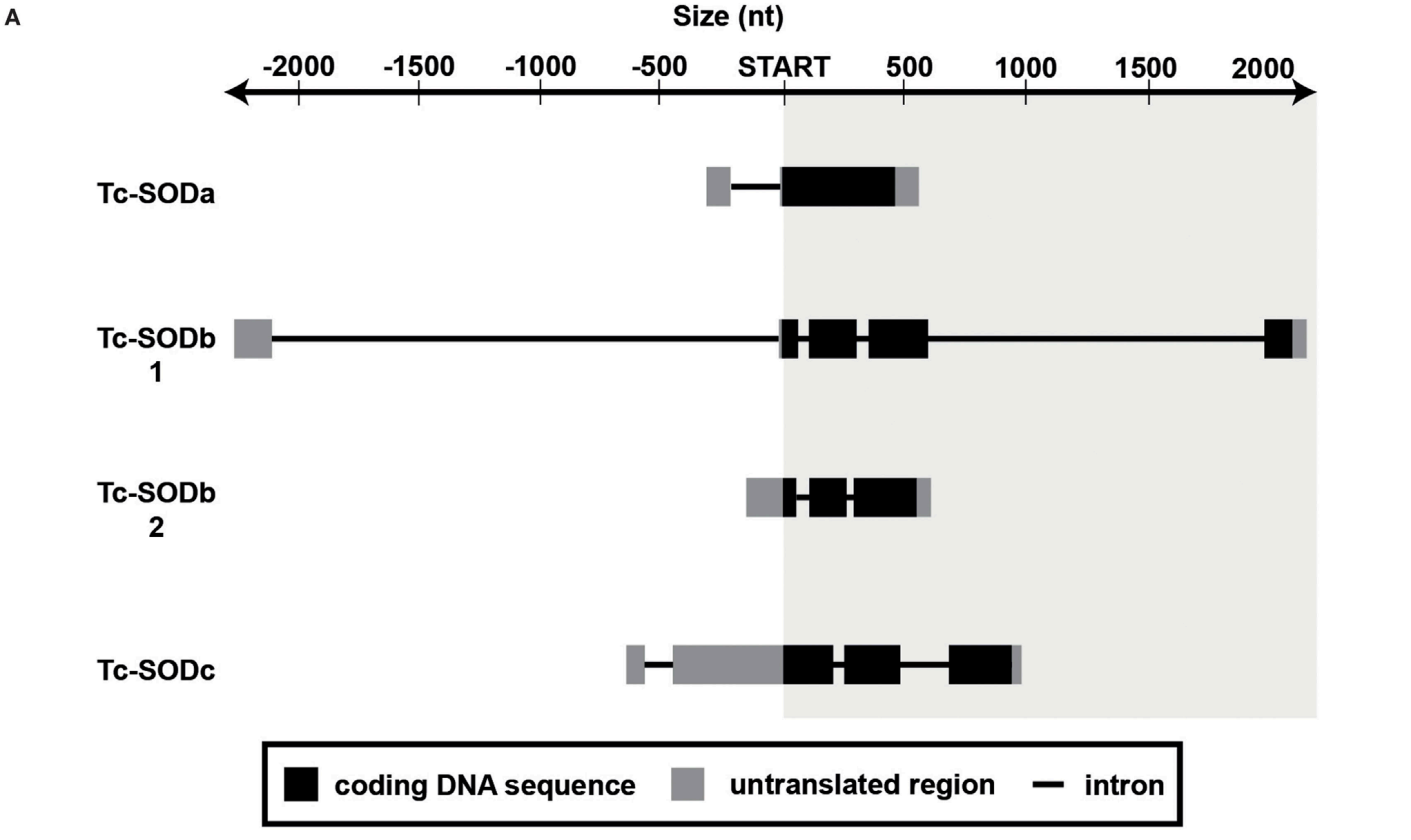

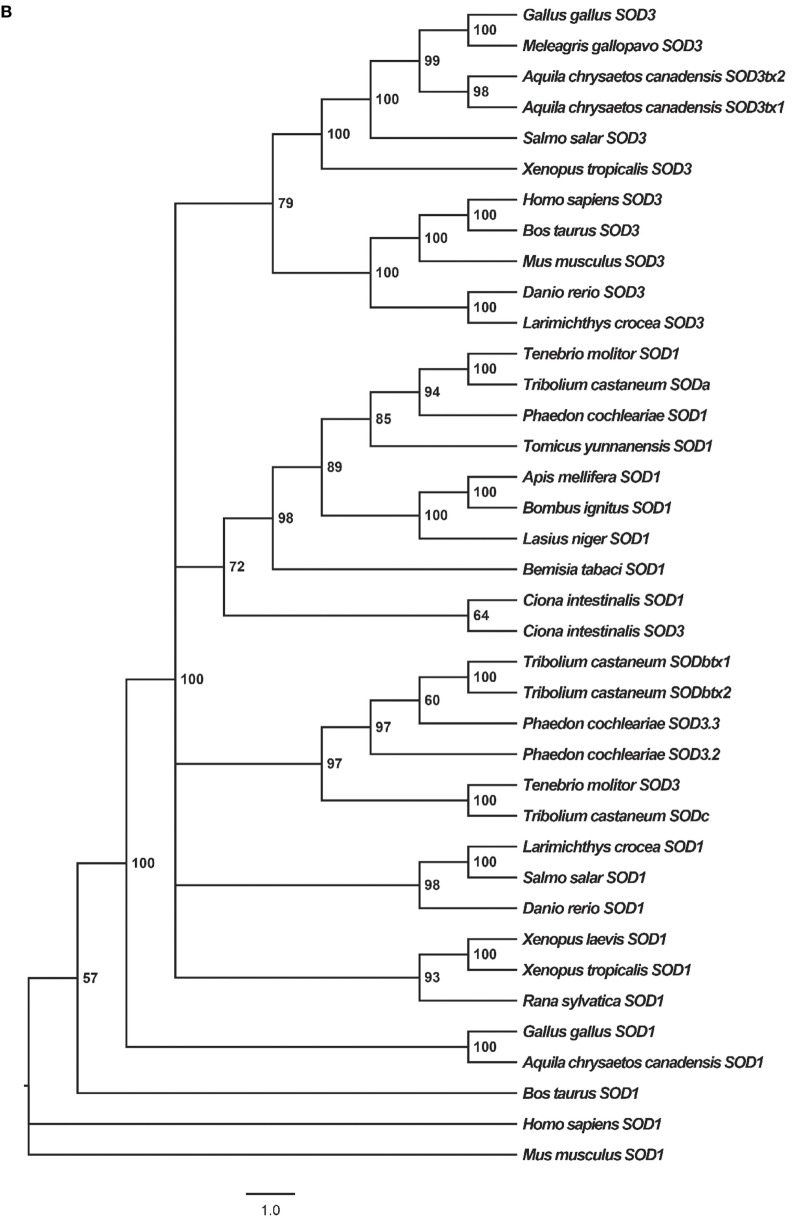
Gene architecture and phylogeny. **(A)** Arrangement of introns and exons in the studied sod isoforms. Exons are depicted as boxes, introns as black lines. For each gene, the coding sequence is indicated in black, the untranslated regions in grey. **(B)** Phylogenetic relationships among *sod1* and *sod3* of various organisms, reconstructed on the basis of cDNA sequences and using Bayesian Inference (BI) (arithmetic mean = −16,649.04; harmonic mean = −16,682.86) method. Posterior probability values higher than 50% are indicated on each node. The scale for branch length (1.0 substitution/site) is shown below the tree. NCBI Reference IDs for the considered sequences are available in Table S2 in Supplementary Material.

*In silico* analysis of *tc-soda* indicates that the open reading frame (ORF) includes 462 nt and that it encodes a putative protein of 155 aa, with a deduced molecular weight of 15.7 kDa. This gene contains two exons and one intron, the latter located in the 5′untranslated (UTR) region (Figure 1A). The length of the 5′- and 3′-UTR are 326 and 100 nt, respectively. The 3′ UTR includes a consensus polyadenylation signal sequence (AATAAA) at nt 879. We identified nine half antioxidant responsive elements (hARE, TGACNNN) putative consensus sequences in both forward and reverse orientation, one full ARE (TGACNNNGC) and one XRE (TGCRCNC) all located in the 1 kb region upstream of the start codon (Table 1).

**Table 1:** Putative responsive elements.

Sequence	ARE		hARE		XRE	
	5′–3′	3′–5′	5′–3′	3′–5′	5′–3′	3′–5′
*soda*	0	1	6	3	1	0
*sodb*	0	0	0	2	0	0
*sodc*	0	0	2	3	0	0

*Summary of the total number and orientation of putative responsive elements in the 1 kb upstream region of tc-soda, tc-sodb, and tc-sodc*.

Integrating information from available RNAseq datasets, we could identify two putative variants for *tc-sodb*, which we named *tc-sodb1* and *tc-sodb2* and that map in the same genomic region. While *tc-sodb1* includes a 615 nt ORF, coding for a 204 aa protein with a deduced molecular weight of 21.4 kDa, *tc-sodb2* includes a 501 nt ORF, coding for a 166 aa protein with a deduced molecular weight of 17.3 kDa. For *tc-sodb1*, the 5′- and 3′-UTR regions are 2,290 and 60 nt long, while the 5′- and 3′-UTR regions for the second transcript are 180 and 59 nt, respectively. The two transcripts display different exon–intron architectures. *tc-sodb1* consists of five exons and four introns, of which the largest one (about 2 kb) is located in the 5′ UTR. *Tc-sodb2* is a truncated form of *tc-sodb1*, consisting of three exons and two introns (Figure 1A). The 3′-UTR region of *tc-sodb1* contains two polyadenylation consensus sequences at 825 and 851 nt, whereas *tc-sodb2* contains only one such site at 721 nt. We identified two hARE sites in the 1 kb upstream of the t*c-sodb genes* (Table 1).

The third identified *sod* gene (*tc-sodc*) is characterised by a 684 nt ORF that encodes a 217 aa protein with a predicted molecular weight of 24.3 kDa. The 5′-UTR region for this gene is 662 nt and the 3′-UTR region is 36 nt long. *tc-sodc* consists of three exons and three introns: one of these introns is located in the 5′ UTR (Figure 1A). We identified one polyadenylation consensus sequence in the 3′ UTR of this gene at 1,239 nt. Our analysis identified two hARE in forward as well as three hARE in reverse orientation in the region upstream of the *tc-sodc* ORF (Table 1).

### Evolutionary Relationship and Molecular Modelling

The deduced amino acid sequences of the four identified Cu,Zn SODs from *T. castaneum* were split into two groups for analyses of the predicted protein structure: *tc-soda* was found to correspond to other metazoan IC-SODs, whereas *tc-sodb1, tc-sodb2*, and *tc-sodc* were found belonging to metazoan EC-SODs. Accordingly, we identified a putative cleavage site for EC-SOD between residues 16 and 17 (TLA-TV) in Tc-SODb1 and 2, as well as between residues 19 and 20 (VQS-AS) in Tc-SODc, but not in Tc-SODa (Figure S1 in Supplementary Material).

Following our initial analysis, the predicted protein sequences of the considered SOD isoforms were aligned with the respective orthologues from other metazoan species with a confirmed crystallography structure (Figure S1 in Supplementary Material). Tc-SODa was compared with SOD1 sequences from *Xenopus laevis* (NCBI NP_001080933.1), *Homo sapiens* (NCBI ABL96616.1), and *Bos taurus* (PDB 1CB4), while Tc-SODb1, Tc-SODb2, and Tc-SODc were compared with SOD3 from *Homo sapiens* only, as this is the only metazoan species with a confirmed crystallographic structure for the EC-SOD (PDB 2JLP). The copper and zinc coordination environment and the active site are conserved in all compared sequences for SOD1 and 3. The 3/10 α-helix in the *C*-terminus is missing from Tc-SODb2 and Tc-SODc.

Figure 1B shows the SOD phylogenetic tree generated by the application of the Bayesian Inference (BI) method to the nucleotide sequences data set, while the respective phylogenetic analysis using amino acid sequences is reported in Figure S2 in Supplementary Material. As expected, both the cDNA and animo acid sequence-based phylogenetic trees group the intracellular isoform *Tc-soda* and extracellular isoform *Tc-sodc* with their respective orthologues from other species in taxonomic order (e.g., *sod1* from the closely related species *T. molitor* forms one clade with our studied isoform). Most intriguing, the two variants of *Tc-sodb* do not fall into the group of *Tenebrionidae*, but seem to be more closely related to the EC-*sod* from the leaf beetle *Phaedon cochleariae* suggesting a gene duplication event of an ancestral EC-*sod* gene before the split of the recent coleopteran families (Figure 1B; Figure S2 in Supplementary Material).

### Cu,Zn-SOD Gene Expression after Immune Priming and Challenge

**Figure 2:**
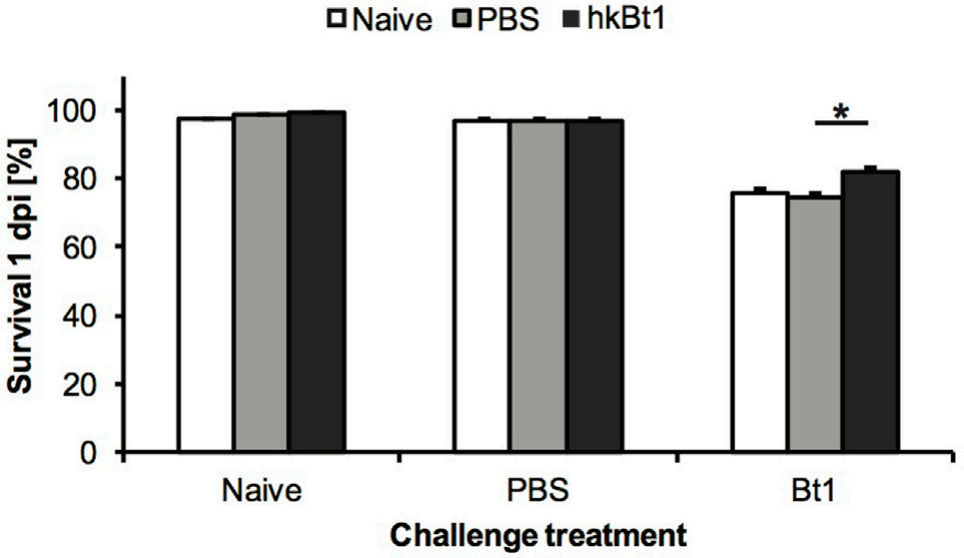
Survival 1-day post-infection (dpi). The proportion of individuals surviving following a challenge when they had been previously primed with heat-killed *Bacillus thuringiensis* [heat-killed Bt1 (hkBt1), dark grey], saline solution (PBS, light grey), or left untreated (Naive, white). Challenge treatments are indicated in the *x*-axis labels: Bt1 = injected with live *B. thuringiensis*, PBS = injected with saline solution, Naive = untreated control. The results are reported as mean of three independent experiments ± SE. Asterisk indicates significant differences within challenge treatments (*p < 0.05).

In order to estimate the contribution of Cu,Zn SOD to immune priming, we conducted a full-factorial priming and challenge experiment following the protocol described in Section “Materials and Methods” (see Animals and Infection Protocol). The primed individuals survived the infection with Bt1 better than both priming control treatments (*p* < 0.05; Figure 2).

**Figure 3:**
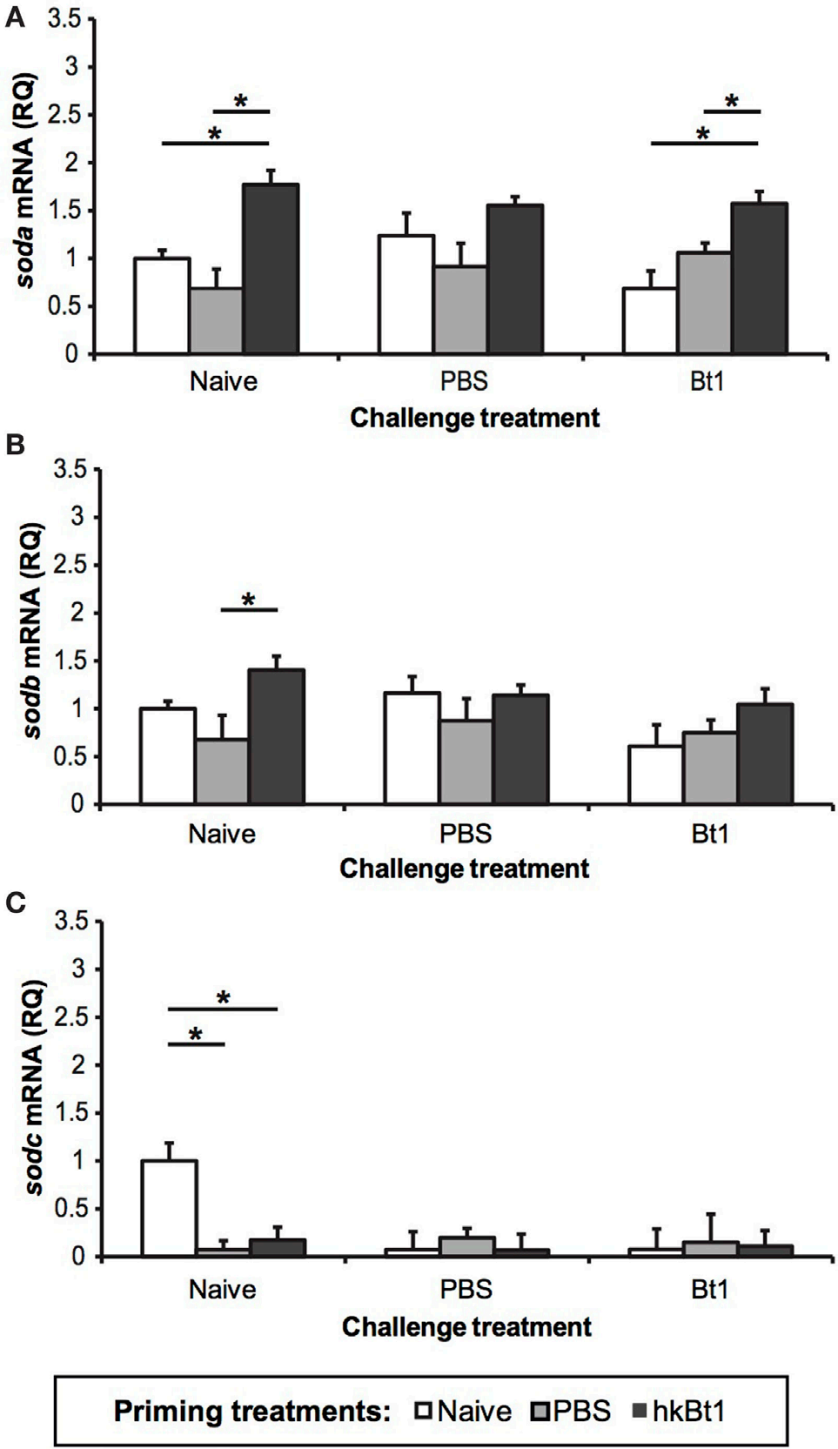
Expression of tc-soda **(A)**, tc-sodb [both transcripts, **(B)**], and tc-sodc **(C)** in all the priming/challenge combinations 1-day post-infection. The results are reported as mean of three independent experiments ± SE. Asterisk: significant differences according to Student–Newman–Keuls *t*-test with respect to priming treatments within challenge treatment (*p* < 0.05).

The Cu,Zn-SOD mRNA levels in all specimens were estimated by RT-qPCR 1 day after the infection. The relative expression of *soda, sodb*, and *sodc* is shown for each priming and challenge combination in Figure 3. *soda mRNA* levels increased for the priming condition when untreated and infected (Figure 3A; *p* < 0.05), but not in the wounding control (priming with PBS), while *sodb* only shows a statistically significant increase of mRNA expression when primed with hkBt1 in the non-challenged treatment, and only if compared with the wounding control (Figure 3B; *p* < 0.05). In contrast, the other gene that codes for SOD3, *sodc*, is down-regulated in all primed or challenged treatments, compared with the untreated control (naive/naive) (Figure 3C; *p* < 0.05). In order to quantify the immune activation due to the priming and/or challenge treatment, we also measured the mRNA expression of the gene for the AMP attacin (*atta2*), which was shown to strongly respond to immune insults. Indeed, atta2 mRNA levels remained low when the animals were unchallenged, but showed increased expression after both repeated wounding and after infection with Bt1 (Figure S3 in Supplementary Material, *p* < 0.05).

**Figure 4:**
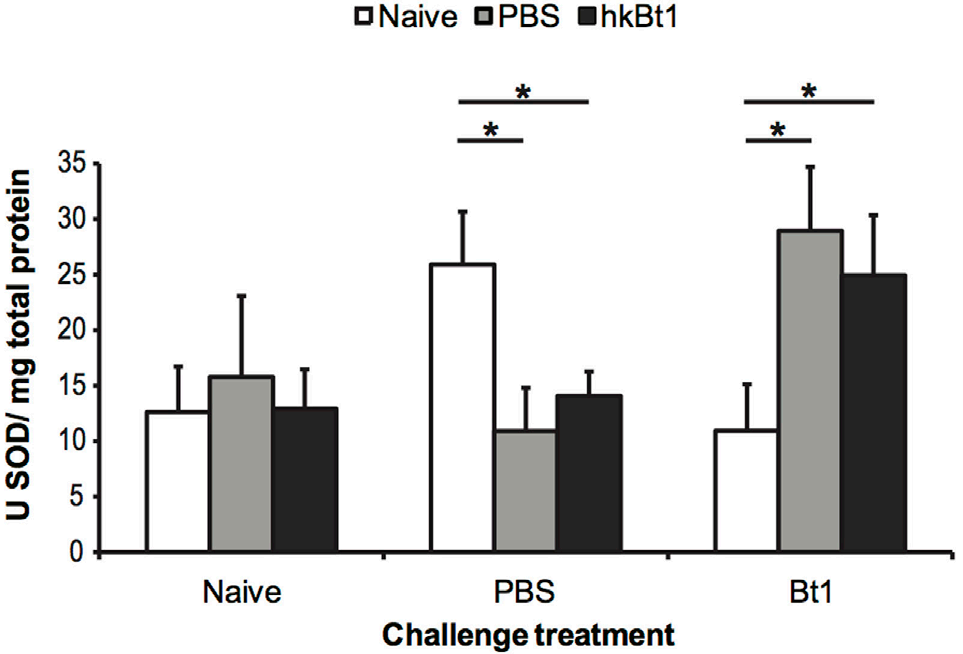
Total superoxide dismutase (SOD) activity 1-day post-infection. The results are reported as mean of three independent experiments ± SE. Asterisk: significant differences according to Student–Newman–Keuls *t*-test with respect to priming treatments within challenge treatment (*p* < 0.05).

The protein activity of SOD remained unchanged in animals without challenge compared with untreated controls (naive/naive) (Figure 4). In PBS challenged specimens, high-SOD activity was only observed in the experimental group that has not been wounded or primed (*p* < 0.05). In infected specimens, the expression pattern of SOD is reversed, resulting in an increase in both wounded and primed experimental groups when compared with the unprimed controls (*p* < 0.05).

## Discussion

In the present study, we comprehensively characterised Cu,Zn-SOD genes in the important pest species *T. castaneum*, which is an intensively used model organism for studies of immune priming, and then experimentally analysed the gene expression of these main components of the antioxidant system. The combination of *in silico* and mRNA expression data demonstrates the presence of one gene coding for IC-SOD (*tc-soda*) and two genes coding for EC-SOD (*tc-sodb* and *tc-sodc*). The two separated genomic regions containing *tc-sodb* and *tc-sodc*, might have resulted from a gene duplication event, that might have contributed to the functional specialisation of these two isoforms in *T. castaneum*, as previously demonstrated in unicellular eukaryotes for IC-SOD and others genes belonging to the antioxidant system (12, 52). In addition, we found that *tc-sodb* occurs in two variants, both of which experimentally confirmed by RTq-PCR (data not shown). The presence of two different mRNAs that map in the same genomic region is not unexpected *per se*. Due to the continued refinement of RNAseq technology, we are now able to identify alternative mRNA transcription, be it as a consequence of an alternative ORF or an intronic alternative polyadenylation recognition [for review see de Klerk and ’t Hoen (53)].

We offer two hypotheses for the redundancy of gene products for EC-SODs in *T. castaneum*. First, as a pest species, *T. castaneum* has been under strong selection pressure by pesticides. Especially, the entomopathogenic bacterium *B. thuringensis* has been used for more than 70 years in agriculture as an efficient bio-insecticide against *T. castaneum*. This constant exposure might have resulted in genotypic variation, which might have contributed to an increased tolerance to pesticides. This might explain our surprising finding of an unusual number of EC-SOD gene transcripts. In fact, as demonstrated in *D. melanogaster*, specific gene duplications can occur in insecticide target sites (e.g., the resistance to dieldrin genes, Rdl), offsetting the beneficial effect of an increased gene product and the negative effect of decreased overall fitness, resulting in a heritable heterozygosity (54). Similar variation in gene copy number of insecticide target sites have been described in aphids (55, 56) and mosquitoes (57). Although a previous study by Contreras et al. (58) found only a minor part of the proteome response of *T. castaneum* larvae to *B. thuringiensis*-derived endotoxins to consist of proteins with predicted antioxidant function, the question remains how representative this finding is of xenobiotic insecticides. While further studies are necessary to answer this question, it can still be argued that EC-SODs (*tc-sodb* and *tc-sodc*) might have been under stronger evolutionary constraints than the intracellular isoform (*tc-soda*), as a consequence of being target sites for insecticides.

A second hypothesis arises from the intriguing finding in our phylogenetic analysis, revealing that the two variants of *tc-sodb* are most closely related to the SOD3 isoforms of the mustard leaf beetle *P. cochlearis*. Interestingly, in this coleopteran species, substantial EC-SOD activity is found in the haemolymph as well as in the defensive secretions, and SOD-deficient larvae showed increased mortality when challenged with the entomopathogenic fungus *Metarhizium anisopliae* (59). Defensive secretions also play an important role in *T. castaneum* for the regulation of the flour microbiota (60, 61), but a potential role of SOD in these fluids needs to be further explored. As the flour microbiota directly contributes to the establishment of the gut microbiota in larvae and because the presence of the gut microbiota in *T. castaneum* is necessary for oral immune priming against *B. t. tenebrionis* (44), regulation of the flour microbiota might constitute a form of social immune priming.

Regarding gene architecture, there are considerable differences across the studied isoforms, not only in gene length but even more in the intron/exon distribution. Since Cu,Zn-SOD evolution is characterised by addition and fusion of exons in regions flanking the catalytic core (62), it is reasonable that the majority of differences were observed in intron distribution and length of the UTR regions. Interestingly, in the gene coding for IC-SOD, which is present in only one copy, only one intron is present, localised in the 5′UTR. This feature might be important in case of erroneous RNA splicing, as the retained intron would not disrupt the protein structure (conserved in *T. castaneum*, see Figure S1 in Supplementary Material) and ultimately the enzyme activity in the cytosol. In the promoter region of all *tc-sod*s, we identified several putative hARE sites, whereas we found one ARE and XRE only in the case of *tc-soda*. Previous studies highlighted that, in invertebrates, a partial ARE sequence is sufficient to promote gene transcription in the presence of ROS (7, 11, 63). Therefore, we can hypothesise that also for *T. castaneum*, full ARE motifs are not required for the ROS-dependent gene transcription. The presence of a large variety and typology of ROS-related putative regulatory elements could confer to Tc-SODa a primary role in the intra-cytoplasmic environmental protection against multiple oxidative stress risk, such as xenobiotic exposure (64).

It has been suggested that ROS might play a role during oral priming responses in *T. castaneum* (38). However, details about infection route dependency and molecular underpinnings, in particular those regarding the potential role of Cu,Zn-SOD genes, were so far lacking. In this work, we have shown for the first time that the priming phenotype in *T. castaneum*, previously obtained by septic pricking (26), can also be induced by haemocoelic injection of *B. thuringiensis* strain DZSM 2046 (*Bt1*). Moreover, we found that *Tc-sod* genes can be induced by such immune insults, potentially through activation *via* the regulatory elements described above. In fact, we detected not only basal transcription of all isoforms in the control specimens (naive/naive), but also a modulation of this transcription in response to septic priming (PBS and hkBt1) and challenge treatments (PBS and live Bt1). The transcription of the IC-SOD (*tc-soda*) was up-regulated after priming with hkBt1 (Figure 3A). This priming response was most visible in animals that were left naive for challenge, but was also still significant in animals that were subsequently challenged with live *B. thuringiensis* (Bt1). Notably, a simple wounding (PBS priming) did not lead to any significant up-regulation of *tc-soda*. In contrast, the priming-induced up-regulation of the transcription of the EC-SOD *tc-sodb* (total mRNA accumulation for transcript one and two), was only visible in animals that were left naive for challenge. Surprisingly, the second EC-SOD (*tc-sodc*) showed a differing pattern of down-regulation in all priming and/or challenge treatments compared with fully naive animals. One potential scenario is that *tc-soda* is up-regulated to protect hosts from the infection-induced •O_2_^−^ formation, while the extracellular isoforms are either down-regulated (*tc-sodc*) or remain mainly transcribed constitutively. This might decelerate the elimination of antimicrobial ROS from the intercellular space (and potentially even in the defensive secretions), while preserving cell viability during infections in *T. castaneum*, ultimately contributing to the overall survival of the host during bacterial infection.

It is noteworthy that the immune-regulatory signal of priming on SOD gene transcription differed from the transcription profile of the AMP gene *atta2*, which mostly showed the expected typical pattern of up-regulation after wounding or bacterial challenge, but did not respond in animals that were primed but left naive for challenge. The role of SOD’s can be viewed as regulators of ROS, and are therefore not directly comparable with immune effector molecules such as AMP’s. This result is in line with what has been reported recently by Tate et al., where *atta2* expression during *B. thuringiensis* infection of *T. castaneum* was highly dependent on bacterial load, while other, metabolic pathways that seem involved in a broader priming effect are not effected by the concentration of bacteria (32, 65). These observations support the view that immune priming represents a specific form of anticipatory response that is different from a simple low-level infection (i.e., challenge) response, as has also been suggested for oral immune priming (38). Unfortunately, our data does not allow for the same level of discrimination between IC- and EC-SOD protein activity, but we confirmed that a priming effect depended on the following challenge: SOD activity was increased in animals that had been primed by both wounding or heat-killed bacteria, but only in those animals, which were later challenged with live bacteria, while this pattern was reversed in animals that received PBS for challenge (Figure 4). While we would have expected a comparatively high level of cumulative SOD activity for the entire Bt1 challenge group, the observed lower SOD activity in the naive/Bt1 group might be the result of an inhibition that could be due to a potential production of H_2_O_2_ during infection. In fact, it is well known that high concentrations of H_2_O_2_ inhibit the enzymatic activity of Cu,Zn SODs *in vivo* (66–68). Such a treatment-specific SOD inactivation in the naive primed group, would imply that the concentration of ROS, and more specifically H_2_O_2_, is an important component of the innate defence mechanism against *B. thuringiensis* in *T. castaneum*. Future studies will allow us to better understand the fate and potential role of H_2_O_2_ produced by SOD activation during immune priming, especially since this metabolite is strongly involved in signalling (69–71) and immunity (14) in vertebrates and invertebrates.

In conclusion, the expression of Cu,Zn-SOD genes is affected by immune priming and challenge in *T. castaneum*. Moreover, our findings support the current idea that priming is a specific reaction that is qualitatively different from a simple low-level immune activation (33, 38) and even involves the overall physiological response. The maintenance of ROS homoeostasis in eukaryotes, is an evolutionary ancient mechanism (72), that is, coadapted as a vital part of innate immunity. This might be another indication that the production of ROS is not only harmful, but can also be desirable to the host (2), especially in an infection scenario. Now that we recognised the potential involvement of the antioxidant system in immune priming, many new questions arise that call for future investigations, e.g., the potential role of peroxide-induced signalling. Indeed, further functional characterisation of the various identified SOD isoforms (as well as other components of the antioxidant system), for example, by RNAi could elucidate their role for immune priming. Finally, we would like to highlight the importance of incorporating both the pre- and post-transcription level of expression into studies of functional genetics for immune-related phenotypes, which will become more prominent in the near future, benefitting from a wealth of recent NGS studies.

## Ethics Statement

This work involved an invertebrate species. No ethical permit is needed according to German law.

## Author Contributions

DF conceived the study. KF, DF, and JK designed the experiments. KF, DF, FC, and GS performed the experiments and assays. DF performed *in silico* characterisation of *sod* genes. RB reconstructed the phylogenetic trees. KF, DF, and GS analysed the data. All authors contributed to writing the manuscript and gave final approval for publication.

## Conflict of Interest Statement

The authors declare that the research was conducted in the absence of any commercial or financial relationships that could be construed as a potential conflict of interest. The reviewer IE and handling editor declared their shared affiliation.

## Footnotes

^1^http://bioinf.uni-greifswald.de/gb2/gbrowse/tcas5/.

^2^http://eu.idtdna.com/analyzer/Applications/OligoAnalyzer/.

^3^http://swissmodel.expasy.org/workspace/.

^4^http://www.R-project.org/.
