# Supplementary material for: Cu,Zn Superoxide Dismutase Genes in Tribolium castaneum: Evolution, Molecular Characterisation, and Gene Expression during Immune Priming
Source: Front Immunol. 2017 Dec 18;8:1811. doi: 10.3389/fimmu.2017.01811 (PMC5763126; doi:10.3389/fimmu.2017.01811)
Supplement: Supplementary file 1 [file Table_1.PDF]

Supplementary material 1: List of qRT-PCR primers with 5'->3' sequences.

Gene name	Primer forward	Primer revers
tc-soda	GAAAGGATCATGGAGGGCC	GTGATTCCAACAACACCACAC
tc-sodb	CTGGCCACAGTGGAGAAAG	GTTGATCACGCCTTCGACTT
tc-sodc	AAACACGGCCTCCACATT	CCATGTGGAGCAGGTAGG
atta2	CAACGACCAAAGGGAAACTA	CTTCCTCCAAGCAAAGTTGG
rp49	TTATGGCAAACTCAAACGCAAC	GGTAGCATGTGCTTCGTTTTG
