# Supplementary material for: Cu,Zn Superoxide Dismutase Genes in Tribolium castaneum: Evolution, Molecular Characterisation, and Gene Expression during Immune Priming
Source: Front Immunol. 2017 Dec 18;8:1811. doi: 10.3389/fimmu.2017.01811 (PMC5763126; doi:10.3389/fimmu.2017.01811)
Supplement: Supplementary file 2 [file Table_2.PDF]

Supplementary material 2: Amino acid and cDNA sequences used for molecular characterisation with NCBI IDs

Species	AA_ID	CDS_ID	isoform
Homo sapiens	NP_000445.1	NM_000454.4	SOD1
Mus musculus	*NP_035564.1*	NM_011434.1	SOD1
Bos taurus	*NP_777040.1*	NM_174615.2	SOD1
Danio rerio	NP_571369.1	NM_131294.1	SOD1
Larimichthys crocea	NP_001290289.1	NM_001303360.1	SOD1
Salmo salar	NP_001117059.1	NM_001123587.1	SOD1
Xenopus laevis	XP_018104357.1	XM_018248868.1	SOD1
Xenopus tropicalis	NP_001016252.1	NM_001016252.2	SOD1
Rana sylvatica	AIT92095.1	KJ847420.1	SOD1
Apis mellifera ligustica	AAR24350.1	AY462419.1	SOD1
Bemisia tabaci	ADO20320.1	HQ230310.1	SOD1
Bombus ignitus	AAZ79896.1	DQ096569.1	SOD1
Phaedon cochleariae	*ALN12448.1*	KT002146.1	SOD1
Phaedon cochleariae	*ALN12447.1*	KT002145.1	SOD3
Phaedon cochleariae	ALN12446.1	KT002144.1	SOD3
Lasius niger	*AAQ81639.1*	AY309973.1	SOD1
Tenebrio molitor	KF527568.1	AII26027.1	SOD1
Tenebrio molitor	AII26026.1	KF527567.1	SOD3
Tomicus yunnanensis	AIW62940.1	KM278528.1	SOD1
Homo sapiens	NP_003093.2	NM_003102.2	SOD3
Mus musculus	AAC62204.1	AF039602.1	SOD3
Bos taurus	NP_001076079.1	NM_001082610.1	SOD3
Danio rerio	XP_001332758.1	XM_001332722.7	SOD3
Larimichthys crocea	NP_001290282.1	NM_001303353.1	SOD3
Salmo salar	NP_001134234.1	NM_001140762.2	SOD3
Xenopus tropicalis	NP_001106630.1	NM_001113159.2	SOD3
Gallus gallus	NP_990395.1	NM_205064.1	SOD1
Gallus gallus	XP_015141186.1	XM_015285700.1	SOD3
Meleagris gallopavo	XP_010708631.1	XM_010710329.2	SOD3
Ciona intestinalis	Ferro *et al.*, 2015	Ferro *et al.*, 2015	SOD1
Ciona intestinalis	Ferro *et al.*, 2015	Ferro *et al.*, 2015	SOD3
Aquila chrysaetos canadensis	XP_011583233.1	XM_011584931.1	SOD3
Aquila chrysaetos canadensis	XP_011583232.1	XM_011584930.1	SOD3
Aquila chrysaetos canadensis	XP_011592069.1	XM_011593767.1	SOD1
Tribolium castaneum	XP_968284.1	-	
Tribolium castaneum	XP_975969	-	
Tribolium castaneum	EFA10685.1	-	
Tribolium castaneum	XP_972244.1	-	
