# Supplementary material for: Cu,Zn Superoxide Dismutase Genes in Tribolium castaneum: Evolution, Molecular Characterisation, and Gene Expression during Immune Priming
Source: Front Immunol. 2017 Dec 18;8:1811. doi: 10.3389/fimmu.2017.01811 (PMC5763126; doi:10.3389/fimmu.2017.01811)
Supplement: Supplementary file 3 [file Data_Sheet_1.pdf]

**Supplementary material 3:** CLUSTAL multiple sequence alignment of intracellular TcSOD a) and extracellular TcSods b) with respective SODs from other metazoan species.

**A)**

```
Tribolium castaneum SODa      MPTKAVCVL--NGEVKGTIFFTQENGKAPVQVTGEVSGLKKGLHGFHIHEFGDNTNGCIS
Xenopus laevis IC-SOD         M-VKAVCVLAGSGDVKGVVHFEQQDE-GAVSVEGKIEGLTDGLHGFHIHVFGDNTNGCMS
Homo sapiens IC-SOD           MATKAVCVLKGDGPVQGIINFEQKESNGPVKVWGSIKGLTEGLHGFHVHEFGDNTAGCTS
Bos taurus IC-SOD             MATKAVCVLKGDGPVQGTIHFEAKGD--TVVVTGSITGLTEGDHGFHVHQFGDNTQGCTS
                              * .******  .* *:* : *  :    .* * *.: **..* ****:* ***** ** *

Tribolium castaneum SODa      AGAHFNPHGKDHGGPTHDVRHVGDLGNIEAGGDGVAKVGITDKFISLEGEHSIIGRTLVV
Xenopus laevis IC-SOD         AGSHFNPENKNHGAPGDTDRHVGDLGNVTAEG-GVAQFKITDSLISLKGPNSIIGRTAVV
Homo sapiens IC-SOD           AGPHFNPLSRKHGGPKDEERHVGDLGNVTADKDGVADVSIEDSVISLSGDHCIIGRTLVV
Bos taurus IC-SOD             AGPHFNPLSKKHGGPKDEERHVGDLGNVTADKNGVAIVDIVDPLISLSGEYSIIGRTMVV
                              **.**** ...**.*    ********: *   *** . * * .***.*  .***** **

Tribolium castaneum SODa      HADPDDLGQGGHELSKTTGNAGARLACGVVGITKA
Xenopus laevis IC-SOD         HEKADDLGKGGNDESLKTGNAGGRLACGVIGYSP-
Homo sapiens IC-SOD           HEKADDLGKGGNEESTKTGNAGSRLACGVIGIAQ-
Bos taurus IC-SOD             HEKPDDLGRGGNEESTKTGNAGSRLACGVIGIAK-
                              * ..****.**:: * .*****.******:* :
```

bold = upper & lower rim

grey highlight = beta sheet, 3/10 helix & alpha helix

dark grey highlight = conserved region for ion binding

**B)**

```
TcSODb1                  MFKLFVLALCATATLATVEKA---------------------------------------
TcSODb2                  MFKLFVLALCATATLATVEKA---------------------------------------
TcSODc                   MFKSVVF-LCTLVTIKNVQSASMQTLAVRNALYQVPGLGNRPLIIKMYPGVENYQSDLYE
Homo_sapiens_EC-SOD      MLAL----LCSCLLLAAGASD-----AWTGEDSAEPNSDSAEWIRDMYAKVTEIWQEVMQ
                         *:      **:   :    .

TcSODb1                  ----------------IVCLKSGD--IDGKITFTQTAEG------VQVEGVINGLPKGKH
TcSODb2                  ----------------IVCLKSGD--IDGKITFTQTAEG------VQVEGVINGLPKGKH
TcSODc                   VYAEPYTFDLRAASAVALIQGEGESAAKGEIVFFQRHPP---NGPILVRGNLTDLPPGKH
Homo_sapiens_EC-SOD      RRDDDGTLHAACQVQPSATLDAAQPRVTGVVLFRQLAPRAKLDAFFALEGFPTEPNSSSR
                                               .:    * : * *          . : *  .    ...

TcSODb1                  GFHIHEKGALGDSCKDAGGHFNPDKKDHGAPEDAVRHVGDLGNIIADDKKVAHVNISDKI
TcSODb2                  GFHIHEKGALGDSCKDAGGHFNPDKKDHGAPEDAVRHVGDLGNIIADDKKVAHVNISDKI
TcSODc                   GLHIHQSGDLRQGCDKLGPHFNPYLLQHGGPSDPVRHVGDLGNIEVEEDGSVEFNIADPL
Homo_sapiens_EC-SOD      AIHVHQFGDLSQGCESTGPHYNPLAVPH------PQHPGDFGNFAVRDGSLWRYRAG--L
                         .:*:*: * * :.*.. * *:**    *      ::*:**:**: . :      . .  :

TcSODb1                  IS-LNGEHSIIGRAVVVHEGEDDLGKGNFNDSKTTGHAGARLVCGVIGIASDGTETCPEG
TcSODb2                  IS-LNGEHSIIGRAVVVHEGEDDLGKGNFNDSKTTGHAGARLVCGVIGIA----------
TcSODc                   LSLMGGPRGIVGRSIVISGNPDDLGRGGTAESLVNGDSGKPIACGVIAYIK---------
Homo_sapiens_EC-SOD      AASLAGPHSIVGRAVVVHAGEDDLGRGGNQASVENGNAGRRLACCVVGVCGPGLWERQAR
                          : : * ..*:**::*:  . ****.*.   *  .* :*  :.* *:.

TcSODb1                  PGNIAISTTGNYFLLFTGLYTLFLNLRF
TcSODb2                  ----------------------------
TcSODc                   ----------------------------
Homo_sapiens_EC-SOD      EHSERKKRRRESECKAA-----------
```

underlined = cleavage site predicted by signalP4.0

bold = upper & lower rim

grey highlight = beta sheet, 3/10 helix & alpha helix

dark grey highlight = conserved region for ion binding
