# Supplementary figures and images for: Cu,Zn Superoxide Dismutase Genes in Tribolium castaneum: Evolution, Molecular Characterisation, and Gene Expression during Immune Priming
Source: Front Immunol. 2017 Dec 18;8:1811. doi: 10.3389/fimmu.2017.01811 (PMC5763126; doi:10.3389/fimmu.2017.01811)

# Supplementary material 4: Phylogenetic analysis of amino acid sequences.

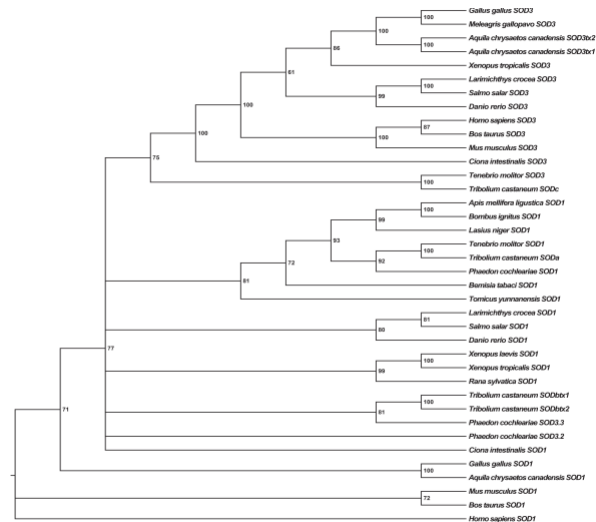

Supplement: Supplementary file 4 [file Data_Sheet_2.pdf]

**Supplementary material 5: attacin 2 gene expression 1 day post infection.**

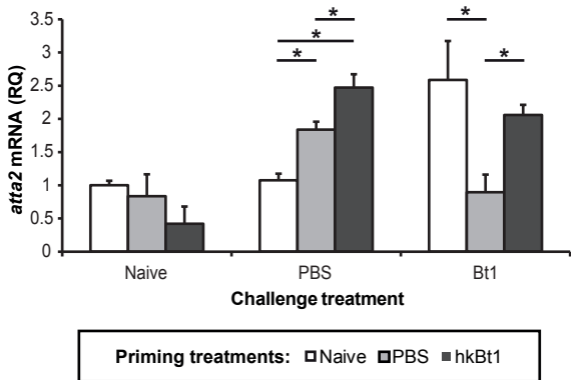

Supplement: Supplementary file 5 [file Data_Sheet_3.pdf]
